# Unique Profile of Proinflammatory Cytokines in Plasma of Drug-Naïve Individuals with Advanced HIV/TB Co-Infection

**DOI:** 10.3390/v15061330

**Published:** 2023-06-06

**Authors:** Marina Nosik, Maria G. Belikova, Konstantin Ryzhov, Darya Avdoshina, Alexandr Sobkin, Vitaly Zverev, Oxana Svitich

**Affiliations:** 1 I.I. Mechnikov Institute of Vaccine and Sera, 105064 Moscow, Russia; rkazaw@yahoo.com (K.R.); vitalyzverev@outlook.com (V.Z.); svitichoa@yandex.ru (O.S.); 2 N.F. Gamaleya National Research Center for Epidemiology and Microbiology, 123098 Moscow, Russia; mariabelikova60@yandex.ru; 3 Chumakov Federal Scientific Center for Research and Development of Immune-and-Biological Products, Russian Academy of Sciences, 108819 Moscow, Russia; darya_avdoshina@mail.ru; 4 Translational Medicine Cluster, Peoples’ Friendship University of Russia, 117198 Moscow, Russia; 5 Department for Treatment of TB Patients with HIV Infection, G.A. Zaharyan Moscow Tuberculosis Clinic, 125466 Moscow, Russia; alexandr@sobkin.net

**Keywords:** HIV-infection, tuberculosis, HIV/TB coinfection, treatment-naïve, proinflammatory cytokines

## Abstract

HIV-1 infection is characterized by aberrant immune activation, and infection with *M. tuberculosis* by an unbalanced production of proinflammatory cytokines. The expression of these cytokines in HIV-1/TB coinfection is still understudied. Here, we aimed to compare the production of proinflammatory cytokines in drug-naive patients coinfected with HIV-1 and *M. tuberculosis* (HIV/TB) compared to patients with respective monoinfections. Plasma samples of patients with HIV/TB coinfection (*n* = 36), HIV-1 monoinfection (*n* = 36), and TB monoinfection (*n* = 35) and healthy donors (*n* = 36) were examined for the levels of eight proinflammatory cytokines. Their levels were significantly increased in all patient groups compared to healthy donors. At the same time, a drastic decrease in the plasma levels of IFN-γ, TNF-α, Il-1β, IL-15, and IL-17 was detected in patients with HIV/TB coinfection compared to patients with HIV-1 or TB monoinfections. The plasma levels of IL-17 characterized the TB severity: in HIV/TB-coinfected patients with disseminated TB, plasma levels of IL-17 were eight times lower than in patients with less severe TB forms (infiltrative TB or TB of intrathoracic lymph nodes; *p* < 0.0001). At the same time, HIV/TB-coinfected patients had increased plasma levels of IL-8, IL-12, and IL-18, with the levels of IL-8 correlating with mortality (*p* < 0.0001). Thus, on the contrary to the patients with HIV-1 or TB monoinfections, HIV/TB-coinfected patients had suppressed production of most of the proinflammatory cytokines associated with antimicrobial immune response, specifically of T-cells involved in the containment of both infections. At the same time, they demonstrated an expansion of proinflammatory cytokines known to originate from both hematopoietic and nonhematopoietic cells, and manifest tissue inflammation. In HIV-1/TB coinfection, this leads to the disruption of granuloma formation, contributing to bacterial dissemination and enhancing morbidity and mortality.

## 1. Introduction

Cytokines are small secreted proteins (<40 kDa) which are produced by nearly every cell to modulate the immune response. The same cytokine can be produced by different cell populations. The effects of cytokines depend on the nature of the targeted cell. Different cytokines may have the same or a synergistic effect. Furthermore, cytokines trigger signaling cascades, giving the smallest amounts of protein the chance to trigger devastating consequences [1,2]. Altogether, these features make cytokines redundant and pleiotropic. Proinflammatory cytokines secreted from immune cells, such as T helper cells (Th) and macrophages as well as nonimmune cells (e.g., keratinocytes and mesenchymal cells), promote inflammation. They are produced immediately after the pathogen enters the body and are involved in the activation and differentiation of immune cells, as well as in the direction of immune cells to the foci of infection in order to control and destroy intracellular pathogens, including viruses [3,4]. The immune response at the initial stages of infection is extremely important; it has a crucial impact on the progression and outcome of the disease as long as it can restrain the development of infection.

At the same time, recent studies have demonstrated that the development of an effective immune response should be balanced, implying the simultaneous concordant release of both pro- and anti-inflammatory cytokines [5]. An unbalanced release of proinflammatory cytokines may lead to a “cytokine storm” [2] similar to the cytokine storm in COVID-19 infection [6]. A systemic inflammatory reaction (acute or chronic) limits the resolution of inflammation and causes disease progression and tissue damage, affecting all organs and systems of the host body [3,7].

Studies have shown that HIV infection is characterized by an aberrant immune activation of all components of the immune system [8,9]. Infection with *M. tuberculosis (Mtb)* also revealed an unbalanced production of cytokines on both local and systemic levels [7,10,11]. A series of studies followed various parameters in HIV-1/tuberculosis (HIV/TB) coinfection or compared to HIV monoinfection to conclude that Mtb and HIV act in synergy, accelerating the decline of immunological functions and leading to subsequent death if untreated [12,13,14]. Multiple studies evaluated the consequences of HIV-1 depletion of T-cells and modified humoral response on the immune responses to *Mtb,* concluding that the mechanisms behind the breakdown of the immune defense of the coinfected individuals are yet not fully understood [15,16,17]. 

Interestingly, direct comparisons of HIV/TB and mono-HIV infection with mono-TB infection are sparse with very little clinical and immunological data [18,19]. Specifically, despite the available clinical evidence that the activation of innate immunity plays a key role in both HIV and tuberculosis (TB) infection, very few studies have been carried out on the expression of proinflammatory cytokines in double infection compared to each of the monoinfections. Hence, there is still no consensus on whether the production of these cytokines in HIV/TB coinfection is suppressed or, conversely, upregulated [12,20]. Our recent study of the expression of pro- and anti-inflammatory cytokines in drug-naïve patients with a dual infection of HIV/TB at the late stages of HIV infection, with previously untreated HIV in the process of receiving antiretroviral (ART) and TB treatment compared to cohorts of patients with HIV and TB monoinfections, revealed an increase in proinflammatory markers such as IL-6, and at the same time, in IL-1Ra [20], which is a natural inhibitor of the proinflammatory effect of IL-1β [21]. 

A characterization of the production of proinflammatory cytokines in patients with HIV/TB would be important for an in-depth understanding of the pathogenesis of coinfection as well as the selection of the effective treatments for associated comorbidities. In this work, we characterized the levels of proinflammatory cytokines in the plasma of drug-naive patients with HIV/TB coinfection compared to treatment-naïve patients with HIV-1 and TB monoinfections; we found cytokine secretion patterns characteristic of each infection form, and show an association between the levels of single proinflammatory cytokines and morbidity and mortality.

## 2. Materials and Methods

### 2.1. Study Population

The subjects were recruited from different population pools at two large medical centers. Patients with dual HIV-1/TB infection (*n* = 36) and patients with TB infection (*n* = 35) were recruited at the G.A. Zaharyan Moscow Tuberculosis Clinic; patients with HIV-1 monoinfection (*n* = 36) were recruited at the Infectious Diseases Clinical Hospital, Moscow, Russia (Table 1). The patients were diagnosed as HIV-seropositive using an ELISA; the ELISA results were confirmed through Western blotting according to the national regulations. TB diagnosis was based on clinical symptoms, sputum microscopy, and radiological analyses. Patients with HIV/TB and HIV patients were naïve for ART, and all patients with TB infection were naïve for antituberculosis therapy. Healthy controls from the general population were recruited for the study at the blood transfusion center. Healthy controls (donors, HDs) repeatedly tested negative for HIV-1 and had no history of TB or exposure to the disease within the past 6 months.

### 2.2. Ethical Statement

All individuals were over 18 years old and gave written informed consent for participation in the study. According to the General Data Protection Regulation (GDPR) requirements, all participants were deidentified and anonymized by assigning them unique codes expressed as an identifier. All clinical samples, data, and study results were stored in an anonymized form. The study was conducted according to the guidelines of the Declaration of Helsinki and approved by the Biomedical Ethics Committee of the I.I. Mechnikov Institute of Vaccines and Sera, Moscow, Russia (protocol #1/01/17/2018).

### 2.3. Sputum Microscopy and Culture

Sputum samples were stained for acid-fast bacilli and were graded with light microscopy. Cultures were examined weekly for a maximum of eight weeks or until positive for visible colonies.

### 2.4. Preparation of Plasma Samples

Plasma was isolated according to the standard procedure. The whole blood was collected in a vacutainer with EDTA and centrifuged at 1000 rpm for 15–20 min with cooling. The plasma was collected, aliquoted, and stored at −80 °C until further analysis. 

### 2.5. Cytokine Quantitation

The plasma levels of IL-1β, IL-8, IL-17, Il-18, IFN-γ, and TNF-α were measured with the enzyme-linked immunosorbent assay (ELISA) using the respective test systems (Vector-Best, Koltzovo, Russia). Tests were performed on undiluted plasma following the manufacturer’s recommendations. The plasma levels of the cytokines IL-12 (p35 subunit) and IL-15 were measured with a Quantikine ELISA Kit (R&D Systems Inc., Minneapolis, MN, USA) as per the manufacturer’s recommendations. Samples were run in duplicates. Cytokine concentrations (pg/mL) were determined using a standard curve obtained with the standards provided by the manufacturer with each kit (with a sensitivity in the range of 0 to 5 pg/mL). The limit of detection for each cytokine is provided in the Supplementary Table S1.

### 2.6. Statistical Analysis

The normality of data distribution was tested using the Kolmogorov–Smirnov test. The nonparametric Mann–Whitney U-test was used to compare two independent groups. The ANOVA test was used to compare three or more independent groups. Dunn’s multiple comparison test was performed to determine the significant differences between the groups. The correlation was evaluated using Spearman’s rank correlation test. The data were analyzed using GraphPad Prism v9.5.0 (GraphPad Software, Boston, MA, USA) and STATISTICA 11.0 software (Tibco, Palo Alto, CA, USA). Multiple regression analysis was performed using the respective module of STATISTICA 11.0. Values of *p* < 0.05 were considered statistically significant. 

## 3. Results

### 3.1. Patients Characteristics

A total of 143 individuals were enrolled in the study: 36 patients with HIV-1/TB coinfection (HIV/TB); 36 patients with HIV-1 monoinfection (HIV); 35 patients with TB monoinfection (TB); and 36 Healthy Donors (HDs). The clinical and demographic characteristics of the patients are presented in Table 1.

The groups did not differ in the age or gender representation of the participants (Table 1). Men predominated in all four study groups. The viral load in patients with double infection was comparable to that in patients with HIV alone. However, there were significant differences in the number of CD4+ cells amongst the patients’ groups (*p* = 0.002). The group of patients with HIV/TB coinfection was dominated by patients with severe immunosuppression (63.9%; CD4+ T-cell counts < 200 cells/mm^3^). In the group of patients with HIV-1 monoinfection, the number of persons with severe immunosuppression was significantly lower (only 19.4% had CD4+ T-cell counts < 200 cells/mm^3^; Table 1). In the period of 6 months after the onset of the therapy, the mortality rate in the group of patients with HIV/TB reached 22.2%. There were no fatal outcomes amongst the patients in the other study groups. 

Coinfections were assessed in patients with HIV/TB coinfection compared to monoinfection. Among the HIV/TB-coinfected patients, 73% (27/36) had other viral coinfections, mainly HBV and HCV (70%, 27/36) and also CMV and HSV. Among the HIV-monoinfected patients, other viral infections were detected in 27% (10/37); 21% (8/37) had viral hepatitis. The difference in the rate of viral coinfections was highly significant (*p* < 0.001, *t* test). 

### 3.2. Plasma Levels of Proinflammatory Cytokines in Patients with HIV-1/TB Coinfection Compared to HIV-1 and TB Monoinfections

The plasma of patients with HIV-1, TB monoinfection, and HIV-1/TB coinfection contained significantly higher levels of all proinflammatory cytokines than the plasma of the healthy donors (Table 2). Furthermore, all three patient groups differed in the levels of most of cytokines, with the major differences observed between the patients with monoinfections and HIV-1/TB coinfection (Table 2, Supplementary Table S1). 

**Figure 1:**
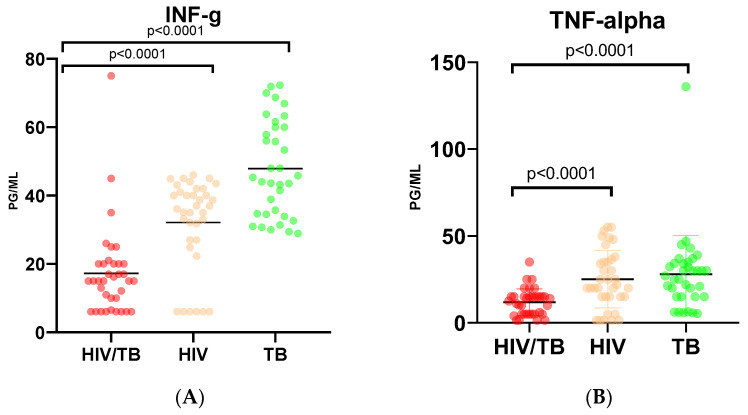

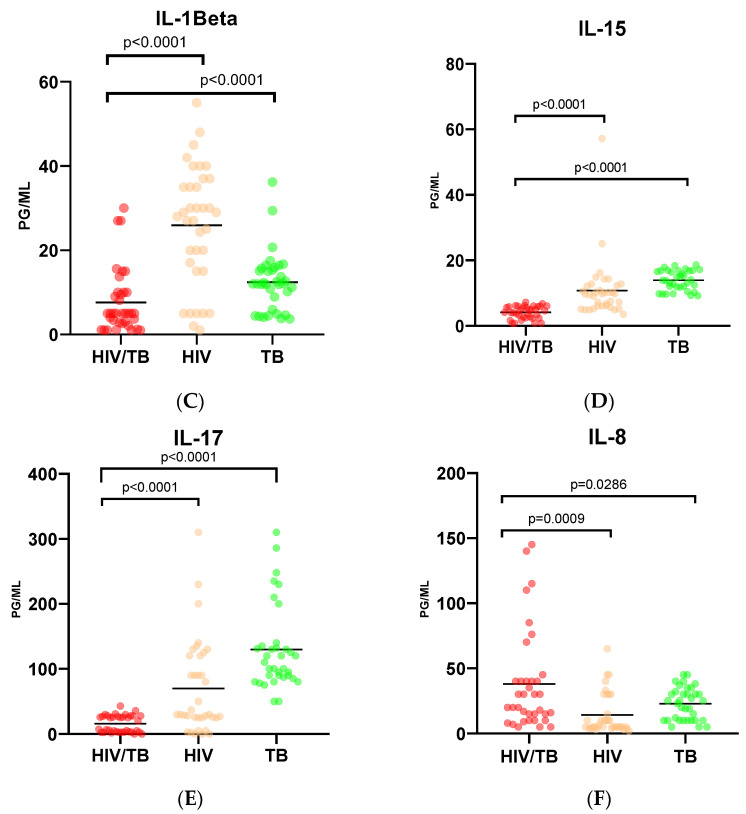

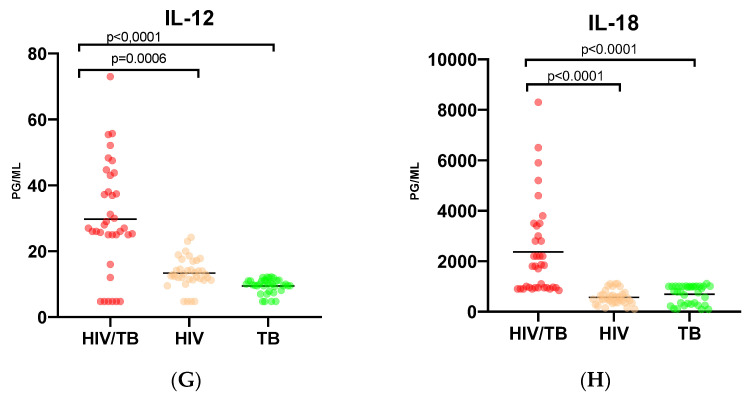
Plasma levels of proinflammatory cytokines in patients with dual HIV/TB infection, HIV-1 monoinfection, and TB monoinfection. Interferon-γ (IFN-g) (**A**), tumor necrosis factor-alpha (TNF-alpha) (**B**), interleukin-1Beta (IL-1Beta) (**C**), interleukin-15 (IL-15) (**D**), interleukin-17 (IL-17) (**E**), interleukin-8 (IL-8) (**F**), interleukin-12 (IL-12) (**G**), and interleukin-18 (IL-18) (**H**). Red circles designate values of the group of HIV/TB co-infected, orange, HIV monoinfected, and green, TB monoinfected patients. Statistical difference: ANOVA test with Dunn’s multiple comparison post-tests; *p*-value < 0.05 was considered significant.

The highest level of the production of INF-γ was observed in the group of patients with TB monoinfection, exceeding the levels observed both in patients with mono-HIV-1 infection and dual HIV-1/TB infection (Figure 1A). HIV/TB patients showed a 1.9-fold decrease in INF-γ production compared to HIV patients and a 2.8-fold decrease compared to TB patients, respectively (*p* < 0.0001). The levels of TNF-α in the HIV-1/TB-coinfected patients were also significantly lower than in the patients with HIV-1 and TB monoinfections (*p* < 0.0001 and *p* < 0.0001, respectively; Figure 1B). The latter two groups did not differ. Furthermore, coinfected patients had significantly lower levels of IL-1β (Figure 1, panel C), IL-15 (Figure 1D), and IL-17 (Figure 1E) than the other two groups. In patients with HIV-1/TB coinfection, the plasma levels of IL-1β were 3.1 times lower than in HIV and 1.5 times lower than in the TB group; those of IL-15 were 2.4 times lower than in HIV and 3.3 times lower than in the TB group; and those of IL-17 were 1.7 times lower than in HIV and 4.2 times lower than in the TB group (all *p* values < 0.0001). Furthermore, among HIV/TB patients, a higher percentage of individuals had undetectable levels of IFN-γ, TNF-α, IL-1β, or IL-15 in plasma compared to the patients with TB monoinfection; the percentage of HIV/TB-coinfected individuals with undetectable levels of IL-1beta or IL-15 was also higher compared to patients with HIV-1 alone (Supplementary Table S1), altogether indicating severe suppression of inflammatory response mediated by hematopoietic cells. 

On the contrary, patients with HIV/TB coinfection had significantly higher levels of IL-8 (Figure 1, panel F), IL-12 (Figure 1G), and IL-18 (Figure 1H) than the other two groups. The difference between mono-HIV- and mono-TB-infected patients was less pronounced: they differed only in four out of eight proinflammatory cytokines, namely: IFN-γ and IL-17, which were lower, and IL-1β and IL-12, which were higher, in HIV-1 compared to the TB-monoinfected patients (Figure 1A,C,E,G). 

In the group of patients with HIV/TB coinfection, IL-8 production was 2.2 times higher compared to the group with HIV alone (*p* = 0.0009) and 2.1 times higher than in patients with TB alone (*p* = 0.0286; Figure 1F). IL-12 production increased by 2.5 times in the group with double infection in comparison with patients with HIV only, and by 1.4 times in comparison with patients with TB only (*p* = 0.0006; Figure 1G). A similar pattern was observed for the production of IL-18. While there was no statistically significant difference in the IL-18 levels between patients with HIV-1 and TB monoinfections, in patients with HIV-1/TB coinfection, the production of this cytokine increased by 4.8 and 4.4 times, respectively (*p* < 0.0001 for both; Table 2 and Figure 1H). Thus, overall, HIV-1/TB-coinfected patients were characterized by decreased or totally abrogated production of INF-γ, TNF-α, IL-1β, IL-15, and IL-17, and hyperproduction of IL-8, IL-12, and IL-18.

**Figure 2:**
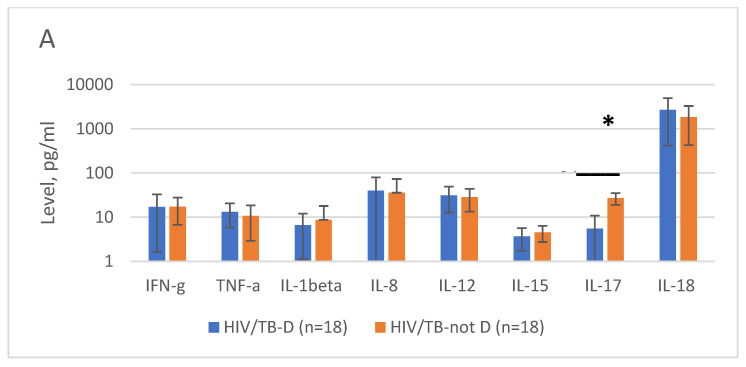

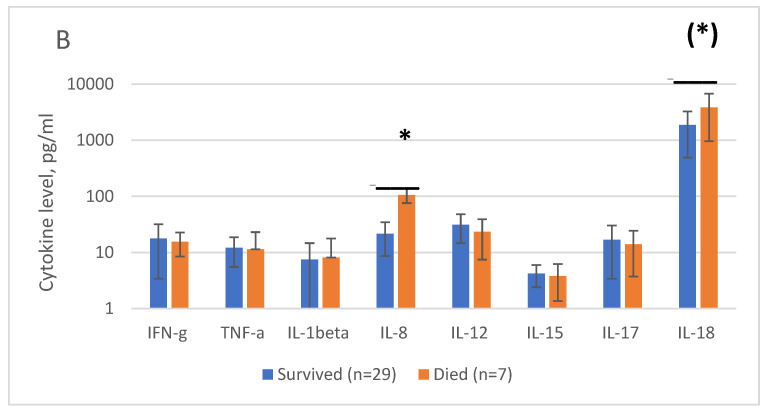
In HIV/TB patients, plasma levels of IL-17 could serve as surrogate markers of morbidity (**A**), and those of IL-8 as predictors of mortality (**B**). Plasma levels of proinflammatory cytokines in HIV-1 patients with disseminated or generalized TB (HIV/TB-D, *n* = 18) versus less severe TB forms (HIV/TB-not D, *n* = 18), *p* = 0.000005 (**A**); Plasma levels of proinflammatory cytokines in HIV/TB patients deceased (*n* = 7) or surviving (*n* = 29) after six months of ART and TB treatment, *p* = 0.0024 (**B**). * *p* < 0.05; (*) *p* < 0.1 in Mann–Whitney U test.

Interestingly, we found that the plasma levels of IL-17 in HIV-1/TB-coinfected patients were associated with the clinical manifestations of TB infection. The plasma level of IL-17 in HIV patients with disseminated or generalized tuberculosis (TB-D) was 8 times lower than in HIV patients with less severe TB forms, and 20 times lower compared to HIV-1-monoinfected patients (*p* < 0.0001 and *p* < 0.0001, respectively) (Figure 2A). The IL-17 levels tended to correlate with patient CD4+ T-cell counts, reflecting the fact that this group was dominated by patients with severe immunosuppression; however, the correlation did not reach the level of significance (R = 0.3198, *p* < 0.1). No difference was found in the levels of other proinflammatory cytokines (Figure 2A). Furthermore, the plasma levels of IL-17 and IFN-γ, but not of the other cytokines nor the CD4+ T-cell counts, served as surrogate markers of the severity of TB manifestations (see Regression Summary for Dependent Variable “TB. Disseminated or not”, *p* = 0.0000000001; Supplementary Table S2).

The IL-8 levels in HIV/TB patients were found to be predictive of mortality. All patients who died within 6 months after the onset of the treatment had four times higher plasma levels of IL-8 compared to the patients that survived, despite all having started the treatment of TB and HIV-1 (Figure 2B). The surviving patients also tended to have higher plasma levels of IFN-γ (Figure 2B). Multiple regression analysis, however, revealed the levels of IL-8 alone to be sufficient predictors of mortality (*p* = 0.0000000000002; Supplementary Table S3).

### 3.3. Correlation of Cytokine Expression with Various Factors and Patterns of Inflammatory Cytokine Secretion Characteristic of the Patient Groups

**Figure 3:**
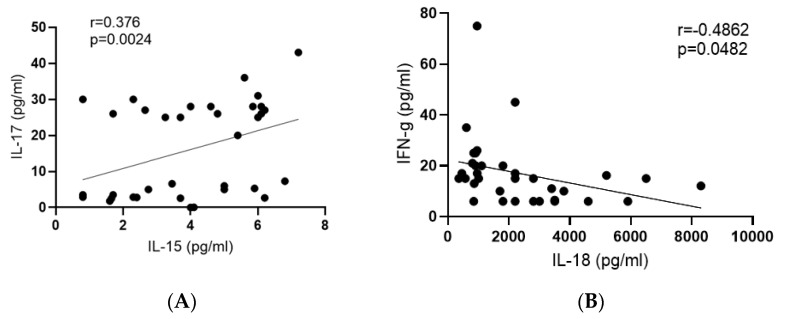
Correlation between concentrations of IL–15 and IL–18 with other cytokines in plasma of patients with HIV/TB. (**A**) positive correlation between plasma levels of IL–15 and IL–17; (**B**) negative correlation between plasma levels of IL–18 and IFN–**γ** (Spearman’s rank correlation test). The r and *p*-values of the correlations are indicated in above the figures. Correlations were considered significant at *p* < 0.05.

Very few significant correlations were found in the plasma levels of INF-*γ*, TNF-α, IL-1β, IL-8, IL-15, IL-17, or IL-18 in any of the groups. However, in the HIV/TB-coinfected patients, we observed a positive correlation between the plasma levels of IL-15 and IL-17 (r = 0.377, *p* = 0.024; Figure 3A) and a negative correlation between the plasma levels of IFN-*γ* and IL-18 (r = *−*0.486, *p* = 0.048; Figure 3B).

We also performed a stringent analysis of the correlations of cytokine levels with CD4+ counts. For all HIV-1-infected patients, proinflammatory cytokine levels were not correlated with CD4+ levels (all *p* values >0.01). The single correlation which was observed to be of relatively high significance (*p* < 0.02) was the correlation of CD4+ counts with the levels of IL-12 (IL-12:CD4+: r = −0.2931, *p* = 0.0125). For the HIV-1-monoinfected patients, the single valid correlation was the inverse correlation of CD4+ counts with the plasma level of IL-18 (CD4+:IL-18: r = −0.6085, *p* = 0.00008). For HIV/TB patients, the cytokine levels were not correlated to the CD4+ counts (or viral load) (all *p* values >0.05). Thus, the CD4+ counts could not explain the observed levels of proinflammatory cytokines within HIV/TB-coinfected patients, nor the difference between the HIV/TB-coinfected and HIV-monoinfected individuals. 

**Figure 4:**
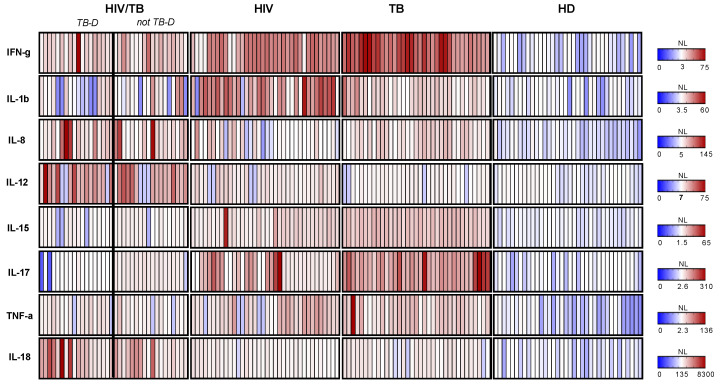
Heat map of cytokine secretion in patients with different infection forms: HIV/TB coinfection with disseminated or generalized TB (HIV/TB, TB-D, *n* = 18) or less severe TB forms (HIV/TB, not TB-D, *n* = 18), HIV-1 monoinfection (HIV, *n* = 36), TB monoinfection (TB, *n* = 35), and healthy donors (HDs, *n* = 36). Cytokines are depicted on the left. Color intensity scale to the right reflects cytokine concentration range.

Despite discordant cytokine levels in the groups, the patterns were very distinctive, indicating the possibility to discriminate the groups by their cytokine secretion profiles (Figure 4).

Indeed, a combination of the levels of secretion of proinflammatory cytokines could distinguish patients with dual HIV/TB and mono-HIV-1 and -TB infections. The plasma levels of five cytokines were significant predictors: IFN-γ, IL-12, IL-15, IL-17, and IL-18 (Table 3).

The IL-8 levels were not predictive; the final model also excluded TNF-α and IL-1β as insignificant (Table 3). Furthermore, a combination of the levels of these five cytokines could distinguish patients with HIV-1 and disseminated or generalized TB (HIV/TB-D) from HIV-1 patients with other forms of TB (HIV/TB-not D), mono-HIV-1, and mono-TB infections (Table 4).

Again, the plasma levels of IFN-γ, IL-12, IL-15, IL-17, and IL-18 were significant predictors. The model excluded IL-1β and IL-8, and the final model also excluded TNF-α (Table 4). The typical “HIV/TB” profile of proinflammatory cytokines included low levels of IFN-γ and IL-15 (two to three times lower than in HIV-1 and TB monoinfections), low IL-17 (5–8 times lower than in both monoinfections), and high levels of IL-12 and IL-18 (3–5 times higher than in both monoinfections) (Figure 1 and Figure 2). 

## 4. Discussion

Both HIV-1 infection, even when successfully treated, and *Mtb* infection are characterized by chronic inflammation. Inflammation is a nonspecific response to pathogens that involves multiple cells, tissues, and organs. While acute inflammation is stopped, chronic inflammation persists, leading to a chronic inflammatory disease characterized by an overproduction of proinflammatory cytokines and recruitment of inflammatory cells (neutrophils and monocytes) to the affected area(s). Blood vessels, enlarged in the acute inflammation to increase blood flow to the sites of infection or trauma, stay permeable. Leukocytes continue to leave the blood and accumulate in the tissue, causing tissue damage and a loss of tissue functions. In the process of persistent immune hyperactivation, cells of the adaptive immune system develop anergy, preventing an effective immune response against new incoming pathogens [22]. This scenario envisages devastating consequences of chronic inflammation to the clinical course of HIV-1/TB coinfection.

*M. tuberculosis (Mtb)* infection is contained in most humans by the immune response dominated by the proinflammatory mechanisms that aim to slow down, sequester, and kill the pathogen. Central to these responses are ROS-generating immune cells, which govern cell death processes, shaping destructive immunity to both *Mtb* infection and TB disease progression [23]. *Mtb* first encounters and infects the alveolar macrophages (AMs) and then additional phagocytic cell types; over time, the bacilli diversify their niche by infecting polymorphonuclear neutrophils (PMNs), DCs, and a variety of tissue-resident and recruited macrophage populations. In the inflammatory environment of the *Mtb*-infected lung, the infected myeloid cells proliferate and differentiate into macrophages and DCs with the proinflammatory phenotype [24]. Interactions between *Mtb* bacilli and host immune agents at the infection site cause chronic granulomatous inflammation [25]. On one hand, it limits the spread of *Mtb*, and on the other, it triggers the M1/M2 polarization of the macrophages with further epithelial mesenchymal transition to epithelioid cells, lipid-rich foamy macrophages, and multinucleated giant cells which act as a *Mtb* niche, inducing tolerance [26,27]. In this way, *Mtb* takes advantage of the proinflammatory environment to survive and further multiply.

In HIV infection as well, persistent inflammation and immune dysfunction are major factors contributing to HIV-associated morbidities, along with non-AIDS events. HIV infection is characterized by a dramatic depletion of CD4+ T-cells, an impaired polarization of Th17 cells, and an inversed balance of gamma delta (γδ) T-cell subsets [22]. Besides the death of CD4+ cells caused directly by HIV-1, one of the factors of progressively diminishing the CD4+ pool is pyroptosis, which is triggered by abortive viral infection [28]. Pyroptosis is characterized by the programmed cell death with the release of proinflammatory cytokines. An important input into the proinflammatory environment is the loss of Th17 cells, one of the major permissive sets of cells for HIV-1 infection. Th17 cells are needed for the maintenance of mucosal barrier integrity and homeostasis, crucial in the defense against fungal and bacterial infections [29]. A loss of Th17 cells in the gut-associated lymphoid tissues leads to the continuous leakage of bacterial triggers of inflammation from the gut [29,30]. Likewise, an important active driver of inflammation in HIV-1 infection is γδ T-cells that express the T-cell receptor consisting of a γ and a δ-chain. In humans, the combination of Vδ gene segments gives rise to two major γδ T-cell populations, Vδ1 and Vδ2. The Vδ1 T-cells form up to 40% of the intraepithelial lymphocytes in the gut, while Vδ2 T-cells constitute the majority γδ T-cells in peripheral blood [31]. HIV-1 infection depletes Vδ2 T-cells, inversing the Vδ2/Vδ1 T-cell ratio in the blood, increasing Vδ1 T-cells [31,32]. The loss of Vδ2 T-cells during untreated HIV disease correlates strongly with CD4+ counts and viral load and occurs quickly after infection [32]. While Vδ2+ T-cells act as Tregs controlling immune activation, Vδ1 T-cells exhibit a distinct proinflammatory cytokine secretion signature [33] and sustain immune activation, even in ART-suppressed patients [34,35].

Thus, *Mtb* and HIV-1 exploit different scenarios of immune hyperactivation/chronic inflammation. Here, we have addressed their overlap in drug-naïve patients with *Mtb* and HIV-1 coinfection, found a downregulation of chronic inflammation, and attempted to dissect the causes and clinical consequences of the altered production of proinflammatory cytokines. Namely, we observed a significant decrease in the production of IFN-γ, TNF-α, IL-1β, IL-15, and IL-17 in patients with HIV/TB coinfection compared to the group of patients with HIV-1 and TB monoinfections. At the same time, coinfected patients demonstrated significantly increased plasma levels of IL-8, IL-12, and IL-18. A combination of the plasma levels of six of these cytokines differentiated the cases of drug-naïve patients at HIV-1 infection stage 3 (CDC classification) with *Mtb* coinfection from the cases of drug-naïve patients at HIV-1 infection stage 3 and drug-naïve patients with *Mtb* monoinfection. Five cytokines further differentiated HIV-1 monoinfection and TB monoinfection. HIV-1/disseminated/generalized TB, and HIV/TB coinfection with less severe TB forms. Interestingly, very few of these cytokine levels were correlated, indicating that the observed pattern(s) resulted from multiparametric (possibly independent) changes in the cytokine production induced by hematopoietic and nonhematopoietic cells. 

IFN-γ is one of the key cytokines for the protection against TB by activating macrophages; it induces the expression of MHC-I and -II, thereby mediating the activation of antigen-presenting cells and the differentiation of CD4 + T-cells [12,36,37]. Cytokines play a critical role in the containment of both HIV-1 and *Mtb* infections. [38,39,40,41]. Here, in agreement with other studies [13], we have shown that patients with TB monoinfection had high plasma levels of IFN-γ, exceeding those in HIV-1 monoinfection, and that HIV/TB coinfection decreased the production of IFN-γ beyond the levels observed in both monoinfections. Furthermore, the decreased plasma level of IFN-γ differentiated HIV/TB coinfection from the respective monoinfections (*p* = 0.0001; Table 2) and, importantly, differentiated coinfections with regard to the severity of clinical manifestations of TB (low levels correlated with severe TB forms, *p* = 0.005; Supplementary Table S2). Both findings pointed to HIV-1 mediating the decrease in plasma levels of IFN-γ, which could be detrimental for the control of TB infection. 

Another proinflammatory cytokine, TNF-α, is a critical regulator of immune responses in healthy organisms and in disease progression that, by triggering different signaling pathways, can lead to both cellular activation and programmed cell death [42]. TNF-α has a primordial function in protection against *Mtb* infection [12,40,43,44,45,46,47]. In HIV-1 infection, TNF-α mediates the apoptosis of HIV-infected cells [48] and suppresses HIV-1 replication in the freshly infected peripheral blood monocytes [49]. Here, corroborating other studies [20,50,51,52,53], we found the plasma levels of TNF-α to be comparable in patients with HIV-1 and with TB monoinfections, but significantly decreased in patients with HIV-1/TB coinfection, although they did not reach significance in distinguishing coinfection from monoinfections (*p* = 0.06), as plasma levels of IFN-γ did. Notably, we found no correlation between the plasma levels of IFN-γ and TNF-α, or any of these cytokines with CD4+ T-cell counts, indicating that the decrease in the levels of both cytokines was due to a loss of different cell populations (or a loss of functionality of different cell populations)— specifically, not the effector CD4+ T-cells. The awaited clinical outcome of the decreased levels of IFN-γ and TNF-α was exemplified by a large percentage of HIV-1/TB-coinfected patients with severe forms of TB observed in this study: almost half of the patients (47.2%) were diagnosed with disseminated and generalized TB, and 25% with infiltrative TB in the decay phase.

In patients with HIV/TB coinfection, we detected low plasma levels of IL-15 as compared to patients with respective monoinfections, which corroborated the results of an earlier study [54]. IL-15 is produced mostly by DCs, macrophages, and epithelial cells [55], as well as by Th17 CD4+ T-cells [56], and regulates proinflammatory T-cell responses [57]. In people living with HIV-1, elevated plasma levels of IL-15 were shown to be a favorable prognostic marker of the outcome of structured interruption of treatment (STI) [58,59]. Likewise, IL-15 plays an important role in protecting against mycobacteria, specifically, in the chronic stage of TB [7,60]. In our study, a decreased plasma level of IL-15 was one of the factors differentiating drug-naïve patients at HIV-1 infection stage 3 coinfected with *Mtb* from the cases of drug-naïve patients at HIV-1 infection stage 3 and drug-naïve patients with *Mtb* monoinfection, supporting an earlier study [61]. However, in this study, plasma levels of IL-15 were not associated with TB severity. This scenario is supported by the data of Queiroz ATL et al., who found that among persons with advanced HIV, the plasma concentration of IL-15 (together with IL-10) identifies TB disease regardless of the time on anti-TB treatment (the treatment reducing TB severity) [61]. Thus, in HIV/TB patients, low plasma levels of IL-15 may reflect a systemic long-term effect of *Mtb* infection on the immune system regardless of the infection course. 

Additionally, our study revealed that patients with HIV-1/TB coinfection have low plasma levels of IL-17 compared to both HIV-1 and TB monoinfections. In HIV-1/TB coinfected patients, decreased plasma levels of IL-17 differentiated cases of drug-naïve patients at HIV-1 stage 3 with *Mtb* coinfection from the cases of drug-naïve patients at HIV-1 stage 3 and patients with *Mtb* monoinfection (*p* = 0.00001). The results of the earlier studies on IL-17 are contradictory. Xu L. et al. showed an increase in IL-17 levels in pulmonary tuberculosis with normalization following TB treatment [62]. Other studies described both low [63] (as in our case) and high levels of this cytokine in the active TB, the latter with the direct correlation between high levels of IL-17 and disease severity [64]. Here, on the contrary to the findings of Jurado J.O. et al. [64], we observed that in HIV-1/TB-coinfected patients, the plasma levels of IL-17 were inversely correlated with the severity of clinical forms of TB. Furthermore, decreased plasma levels of IL-17, together with low levels of IFN-γ, differentiated HIV-1/disseminated/generalized TB from HIV-1/TB coinfection with less severe TB forms (*p* = 0.00000000001; Supplementary Table S2). A sharp decrease in the plasma levels of IL-17 could have been associated with the decrease in the population of IL-17-secreting (Th17) CD4+ T-cells characteristic of both TB [65] and HIV-1 infections [66,67]. 

Interesting in this context is one of the rare correlations found in this study—a significant (*p* = 0.002) correlation of plasma levels of IL-17 and IL-15 observed in patients with HIV-1/TB coinfection, but not in either of the monoinfections. In healthy individuals, IL-15 produced by innate immune cells downregulates the production of IL-17 by T-cells [56]. Chronic autoimmune diseases, on the contrary, are characterized by a direct correlation between the levels of IL-15 and IL-17, as was shown for idiopathic inflammatory myopathies, rheumatoid arthritis (concomitant with an increase in the levels of IL-18), and multiple sclerosis [57,68,69], as well as in the wide range of autoimmune and systemic diseases associated with HIV-1 infection [70,71,72]. The concordantly reduced plasma levels of IL-17 observed here in HIV-1/TB coinfection can, thus, be interpreted as a marker of chronic inflammation. This phenomenon calls for further in-depth mechanistic studies. 

Among the plasma levels of inflammatory cytokines, the levels of IL-1β were the least specific/selective for HIV-1/TB coinfection. IL-1β is a member of the interleukin-1 family of cytokines produced by inflammatory cells of myeloid lineage, mainly activated macrophages [73]. IL-1β is necessary for the control of mycobacterial infection [74], patients with TB are characterized by increased plasma levels of IL-1β, especially in the cases of bilateral breakdown of lung tissue [75]. This coincides with the results of this study, where an increased level of IL-1β secretion was detected in 75% of patients with TB monoinfection. In HIV-1, IL-1β induces the production of adhesion molecules that promote cell-to-cell contact and the transendothelial migration of inflammatory cells, promoting the spread of HIV target cells and favoring viral replication [76]. Here, in agreement with earlier studies [77,78], patients with HIV-1 monoinfection exhibited increased plasma levels of IL-1β, two-fold higher than in patients with TB, and six-fold higher than in healthy individuals. However, in HIV-1/TB coinfection, we found the level of IL-1β production to be significantly reduced compared to both monoinfections, even though 28.6% of HIV/TB patients were diagnosed with the TB-induced destruction of lung tissues. These findings point to a severe depletion and/or nonfunctionality of IL-1β-producing cells (macrophages) in HIV/TB-coinfection. 

To conclude, in HIV-1/TB-coinfected patients, we observed a significant decrease in the plasma levels of proinflammatory cytokines produced by hematopoietic cells, macrophages, and CD4+ T-cells in comparison to patients with HIV-1 and TB monoinfections. The insufficient production of these cytokines leads to the limited recruitment of monocytes to the sites of development of pathological processes in HIV infection, thereby contributing to chronic inflammation. In TB infection, reduced expression of proinflammatory cytokines leads to the disruption of the granuloma formation process, as a result of which a defective granuloma is formed and *Mtb* dissemination occurs, which in turn leads to an aggravation of the clinical course of TB infection. 

Against the background of reduced levels of IFN-γ, TNF-α, IL-15, IL-17, and IL-1β, we observed the hyperproduction of IL-8, IL-12, and IL-18. IL-8 plays a central role in the recruitment of T-lymphocytes into the granuloma and their activation inside it [79]. However, at the same time, the increased expression of IL-8 observed in HIV infection contributes to the protection of HIV-infected cells from death, leading to an expansion of infection [80,81]. Of note, IL-8 is secreted not only by the immune cells, such as monocytes and neutrophils, but also by the epithelial, fibroblast, endothelial, and mesothelial cells, manifesting chronic tissue inflammation [82]. The latest studies on COVID-19 demonstrated the association of high IL-8 levels with respiratory failure and poor survival [83]. Likewise, here, we found an increase in the number of deaths in a subgroup of HIV/TB patients with high IL-8 levels compared to a subgroup of HIV/TB patients with low IL-8 levels. Further studies are needed to define if this cytokine can serve as a potential biomarker of the disease outcome in HIV/TB coinfection. 

Another proinflammatory cytokine, IL-12, mediates protective immunity against both HIV-1 and *Mtb*, specifically at the initial stage of infection [44]. IL-12 is involved in granuloma formation by promoting the Th1 response and inducing IFN-γ-positive CD4 T-cells [84,85,86]. Interestingly, in HIV-1 infection, even successfully treated patients demonstrate elevated levels of IL-12 [87,88,89], concomitant with a blunting of IL-12-induced in vivo CD4+ T-cell activation [90]. In our study, the plasma levels of IL-12 in HIV/TB coinfection significantly exceeded those in HIV monoinfection, while in TB monoinfection, IL-12 levels did not differ from those in healthy donors (Table 2). The production of IL-12 by phagocytic cells is induced by both T-cell-dependent and T-cell-independent mechanisms [91,92]. The T-cell-independent mechanism is induced by bacteria/bacterial products [91,92]. Of note, IL-12 is a heterodimer composed of p40 and p35 subunits. The test system used in this study detected the p35 subunit of IL-12. While p40 is solely produced by T-cells, p35 is also produced by nonhematopoietic (radiation-insensitive) cells [93]. Furthermore, we found that the plasma levels of IL-12 (p35 subunit) were correlated with CD4+ counts only in HIV-1 monoinfection, not in HIV/TB coinfection. Altogether, these data indicate an *Mtb-*stimulated hyperexpression of the p35 subunit of IL-12 by non-T-cells. Interestingly, the plasma levels of IL-12 differentiated HIV/TB coinfection from HIV-1 and TB monoinfections (Table 3 and Table 4), but could not differentiate between severe and less severe forms of TB (Figure 2A; *p* = 0.29, Table 4; Supplementary Table S2). Altogether, this pointed to the role of HIV-1, not *Mtb*, in the dysregulation of IL-12 production with the involvement of nonhematopoietic cells. Which nonhematopoietic cells are involved and what input they bring into the pathology of HIV-1 and HIV/TB coinfection remain to be elucidated. 

Last but not least, we demonstrated an overproduction in HIV/TB patients of IL-18. IL-18 activates CD8+ T-cells, which play a central role in the protective immunity against *Mtb* infection [94,95]. At the same time, it induces HIV-1 replication, which contributes to aberrant immune activation [94,96]. Increased IL-18 secretion is associated with a sharp decrease in the number of CD4+ cells and fast progression of the disease [77,97,98,99]. Excessive production of IL-18 can cause local or systemic damage to the body [94,100]. Over-expression of IL-18 is tightly associated with intestinal inflammation and decreased intestinal integrity as measured by intestinal permeability, regeneration, and repair [101]. In our study, HIV-1/TB coinfection was characterized by extremely high plasma levels of IL-18, which were increased almost five-fold compared to the levels in patients with HIV-1 and TB monoinfections. The levels of IL-18 differentiated cases of coinfection from both HIV-1 and TB monoinfections (*p* = 0.015, Table 2), indicating the additive effects of two pathogens on IL-18 production. An inverse correlation was observed between plasma levels of IL-18 and IFN-γ, reflecting the synergistic effects of HIV-1 and *Mtb* in immune suppression. In this way, *Mtb*-induced production of IL-18, protective for *Mtb* infection, appeared to be detrimental for HIV-1/TB coinfection, contributing to HIV-1 persistence and tissue damage. 

This disbalance of proinflammatory cytokine production could be attributed to the HIV-1-induced loss of CD4+ T-cells, specifically Th17 cells and gamma delta T-cells, which play a critical role in the control (containment and elimination) of *Mtb*, altogether resulting in aggravation of the clinical course of TB infection in HIV-1-infected individuals. The specific mechanisms behind this disbalance, specifically the nature of the affected hematopoietic cells, remain to be elucidated. 

## 5. Conclusions

In summary, we have shown that patients with HIV-1/TB coinfection are characterized by reduced plasma levels of five proinflammatory cytokines: IFN-γ, TNF-α, Il-1β, IL-15, and IL-17, which are crucial for the control of both infections. At the same time, HIV/TB-coinfected patients demonstrate a hyperproduction of IL-8, IL-12, and IL-18, shown earlier to enhance HIV-1 replication and reported to support inflammation of the epithelial tissues. We are aware of the limitations of this work associated with the small sample of patients. Future studies will be conducted on a wider group of patients and will include a detailed characterization of the immune status of the patients, including typing of the cytokine-producing cells, to attribute the observed profiles of cytokine expression as well as to correlate it with the clinical course of both infections.

## Figures and Tables

**Table 1:** Baseline demographic and clinical characteristics of study population.

Characteristics	HIV/TB (*n* = 36)	HIV (*n* = 36)	TB (*n* = 35)	Healthy Donors (*n* = 36)
**Gender, (n/%):**				
**Male**	23 (63.9)	24 (66.7)	25 (71.4)	27 (75)
**Female**	13 (36.1)	12 (33.3)	10 (28.6)	9 (25)
**Age (years), IQR**				
**Male**	36.0 (27–51)	35.8 (26–52)	36.9 (28–65)	34.9 (22–42)
**Female**	34.8 (26–52)	35.8 (21–50)	34.5 (26–72)	34.2 (22–38)
**TB forms, (*n*/%):**				
**Disseminated,** **Generalized**	17 (47.2)		7 (20)	
**Infiltrative**	9 (25)		27 (77.1)	
**TB of intrathoracic lymph nodes**	10 (27.8)	-	1 (2.9)	-
**CD4+ count (%/cells/mm^3^), IQR:**	292	314		
**<200**	63.9%/52 (1–187)	19.4%/99 (17–198)		
**<350**	16.7%/274 (221–341)	66.7%/292 (248–342)		
**>350**	19.4%/549 (386–740)	13.8%/552 (420–745)	499 (368–570)	984 (800–1350)
**Viral load (log_10_ copies/mL), IQR**	5.85 (2.72–6.78)	5.67 (1.9–6.6)	-	-

IQR—interquartile range.

**Table 2:** Levels of cytokines in the plasma of patients with HIV-1, TB, and mixed HIV-1/TB infection compared to healthy donors. Values represent Mean ± SEM. Statistical difference was assessed using F-test (Statistica).

Cytokine, pg/mL	Healthy Donors (*n* = 36)	HIV/TB (*n* = 36)	HIV (*n* = 36)	TB (*n* = 35)
**IFN-γ**	**2.8 ± 1.1 ^a^**	**17.2 ± 13.1 ^b,c^**	**32.1 ± 13.2 ^d^**	**47.9 ± 14**
**TNF-α**	**2.3 ± 1.5 ^a^**	**11.95 ± 7.6 ^b,c^**	25 ± 16.5	27.9 ±22.3
**IL-1β**	**3.5 ±1.2 ^a^**	**7.6 ± 5.6 ^b,c^**	**25.9 ± 14 ^d^**	**12.4 ± 6.9**
**IL-8**	**5.5 ± 2.3 ^a^**	**44.1 ± 31.9 ^b,c^**	20.4 ±15.6	21.4 ± 12.3
**IL-12**	**8.06 ± 1.2 ^a^**	**29.7 ± 16.5 ^b,c^**	**13.2 ± 4.6 ^d^**	**9.4 ± 2.2**
**IL-15**	**1.5 ± 0.4 ^a^**	**4.1 ± 1.9 ^b,c^**	10.8 ±8.9	13.9 ± 2.9
**IL-17**	**2.6 ± 0.7 ^a^**	**16.3 ± 12.5 ^b,c^**	**69.8 ± 55.4 ^d^**	**129.6 ± 65.01**
**IL-18**	**141.9 ± 43.9 ^a^**	**3086.7 ± 1901.7 ^b,c^**	639.8 ± 278.09	694.6 ± 361.2

^a^ Difference between HDs and all patient groups, *p* < 0.05; ^b^ Difference between HIV/TB patients and patients with HIV-1 monoinfection, *p* < 0.0001; ^c^ Difference between HIV/TB patients and patients with TB monoinfection, *p* < 0.0001; ^d^ Difference between HIV-1 monoinfected patients and patients with TB monoinfection, *p* < 0.005.

**Table 3:** Distinct profiles of proinflammatory cytokine production in patients with dual HIV/TB and mono-HIV-1 and -TB infections through multiple linear regression analysis.

*n* = 107	b*	Standard Error of b*	b	Standard Error of b	T(99)	*p*-Value
Intercept			101.4531	0.177180	572.5980	0.000000
IFN-g	0.256601	0.064765	0.0115	0.002898	3.9620	0.000140
IL-12	−0.246967	0.060972	−0.0152	0.003745	−4.0505	0.000102
IL-17	0.278482	0.060229	0.0031	0.000678	4.6237	0.000011
IL-15	0.243979	0.059068	0.0289	0.006994	4.1304	0.000076
IL-18	−0.145598	0.058582	−0.0001	0.000035	−2.4854	0.014618
TNF-a	0.101528	0.053948	0.0047	0.002474	1.8820	0.062777
IL-1beta	−0.060877	0.055109	−0.0039	0.003555	−1.1047	0.271976

Regression Summary for Dependent Variables “Patient group” (HIV, TB, and HIV/TB infection): R = 0.86722103; R^2^ = 0.75207232; Adjusted R^2^ = 0.73454208; F(7,99) = 42.901; *p* < 0.0000 Std. Error of estimate: 0.42164.

**Table 4:** Distinct profiles of proinflammatory cytokine production in patients with HIV-1 and disseminated or generalized TB (HIV/TB-D), or other forms of TB (HIV/TB-not D), TB, and mono-HIV-1 and mono-TB infections through multiple linear regression analysis.

*n* = 107	B*	Standard Error of b*	b	Standard Error of b	T(99)	*p*-Value
Intercept			102.1979	0.215129	475.0531	0.000000
IL-17	0.412481	0.056526	0.0066	0.000908	7.2971	0.000000
IL-15	0.218270	0.055102	0.0369	0.009313	3.9612	0.000140
IL-12	−0.219363	0.056528	−0.0192	0.004956	−3.8806	0.000187
IL-18	−0.191104	0.053887	−0.0002	0.000046	−3.5464	0.000596
IFN-g	0.158347	0.060543	0.0101	0.003868	2.6154	0.010291
TNF-a	0.053577	0.050432	0.0035	0.003302	1.0624	0.290629

Regression Summary for Dependent Variables “Patient group detailed” (HIV, TB, HIV/TB-D, HIV/TB-not D): R = 0.88149533, R^2^ = 0.77703402, Adjusted R^2^ = 0.76365606, F(6,100) = 58.083, *p* < 0.0000 Std. Error of estimate: 0.56792.

## Data Availability

Data available upon request from the authors.

## Supplementary Materials

The following supporting information can be downloaded at: https://www.mdpi.com/article/10.3390/v15061330/s1, Table S1: Representation of individuals with undetectable levels of proinflammatory cytokines among patients with acute HIV, TB, and HIV/TB infections. Table S2: Levels of cytokines in the plasma of patients with HIV-1, TB, and mixed HIV-1/TB infection compared to healthy donors. Table S3: Survival of treatment-naive patients with HIV/TB coinfection (*n* = 36) in six months after the onset of treatment can be predicted by the plasma levels of IL-8.

## Abbreviations

ART	Antiretroviral therapy
IFN-γ	Interferon-γ
IL-1β	Interleukin-1 β
IL1Ra	Interleukin-1 receptor antagonist
IL-8	Interleukin-8
IL-12	Interleukin-12
IL-15	Interleukin-15
IL-17	Interleukin-17
IL-18	Interleukin-18
TNF-α	Tumor necrosis factor-α
HIV/TB	Human immunodeficiency virus/tuberculosis
Mbt	Mycobacterium tuberculosis
TB	Tuberculosis
ELISA	Enzyme-linked immunosorbent assay
