# Supplementary material for: Unique Profile of Proinflammatory Cytokines in Plasma of Drug-Naïve Individuals with Advanced HIV/TB Co-Infection
Source: Viruses. 2023 Jun 6;15(6):1330. doi: 10.3390/v15061330 (PMC10303992; doi:10.3390/v15061330)
Supplement: Supplementary file 1 [file viruses-15-01330-s001.zip › viruses-2316344-Supplementary.pdf]

Supplementary Table S1. Representation of individuals with undetectable levels of proinflammatory cytokines among patients with acute HIV, TB and HIV/TB infections.

Cytokine	Limit of detection (pg/ml)	Patients with HIV/TB (n=36)		Patients with HIV (n=36)		Patients with TB (n=35)		Percentage deviation
		n	%	n	%	n	%	
**IFN-g**	6.05	8	22.2 *p=0.003*[a]	6	16.7 *p=0.003*[b]	0	0	0.33
**TNF-α**	1.5	4	11.1 *p=0.04*[a]	5	13.8 *p=0.003*[b]	0	0	
**IL-1Beta**	1.05	4	11.1 *p=0.043*[a]	1	2.8	0	0	3
**IL-8**	4	0	0	0	0	0	0	
**IL-12**	4.75	6	16.7	4	11.1	4	11.4	0.5
**IL-15**	3.8	4	11.1 *p=0.043*[a]	1	1	0	0	3
**IL-17**	2.9	0	0	0	0	0	0	
**IL-18**	92.5	0	0	0	0	0	0	

If undetectable, cytokine levels were replaced by the value equal to half of the lower limit of detection for cytokine in question, according to the instructions of the manufacturer. [a] – p value for the difference between HIV/TB and TB patient groups; [b] – p value for the difference between HIV and TB patient groups in comparison by F-test (STATISTICA). No difference was revealed in other pair-wise comparisons.

Supplemental Table S2. Severity of TB manifestations in HIV/TB co-infected patients (n=36) can be predicted by the plasma levels of IL-17 and IFN-g.

N=36	b*	Standard error of b*	b	Standard error of b	T(99)	p-value
Intercept			100.9711	0.115078	877.4134	0.000000
IL-17	0.914574	0.083975	0.0354	0.003253	10.8910	0.000000
IFN-g	−0.244292	0.081142	−0.0095	0.003140	−3.0107	0.005148
CD4+	0.109803	0.081067	0.0003	0.000195	1.3545	0.185379
IL-12	0.086330	0.079973	0.0026	0.002444	1.0795	0.288694

Regression Summary for Dependent Variable "TB Disseminated or not": R= 0.90062486; R^2= 0.81112514; Adjusted R^2= 0.78675419; F(4,31)=33.282 p=0.0000000000819138507; Std.Error of estimate: 0.23417; Include cases: 1:36

Supplemental Table S3. Survival of drug naive patients with HIV/TB co-infection (n=36) in six months after the on-start treatment can be predicted by the plasma levels of IL-8.

N=36	b*	Standard error of b*	b	Standard error of b	T(99)	p-value
Intercept			−8577.60	741.8936	−11.5618	0.000000
Death in 6 mths after ART	0.893673	0.076955	84.31	7.2596	11.6130	0.000000

Regression Summary for Dependent Variable"IL-8": R=0.89367324 R^2= .79865187; Adjusted R^2= 0.79272986; F(1,34)=134.86; p=0.00000000000221791796; Std.Error of estimate: 17.239; Include cases: 1:36
